# Physiological parameters for Prognosis in Abdominal Sepsis (PIPAS) Study: a WSES observational study

**DOI:** 10.1186/s13017-019-0253-2

**Published:** 2019-07-15

**Authors:** Massimo Sartelli, Fikri M. Abu-Zidan, Francesco M. Labricciosa, Yoram Kluger, Federico Coccolini, Luca Ansaloni, Ari Leppäniemi, Andrew W. Kirkpatrick, Matti Tolonen, Cristian Tranà, Jean-Marc Regimbeau, Timothy Hardcastle, Renol M. Koshy, Ashraf Abbas, Ulaş Aday, A. R. K. Adesunkanmi, Adesina Ajibade, Lali Akhmeteli, Emrah Akın, Nezih Akkapulu, Alhenouf Alotaibi, Fatih Altintoprak, Dimitrios Anyfantakis, Boyko Atanasov, Goran Augustin, Constança Azevedo, Miklosh Bala, Dimitrios Balalis, Oussama Baraket, Suman Baral, Or Barkai, Marcelo Beltran, Roberto Bini, Konstantinos Bouliaris, Ana B. Caballero, Valentin Calu, Marco Catani, Marco Ceresoli, Vasileios Charalampakis, Asri Che Jusoh, Massimo Chiarugi, Nicola Cillara, Raquel Cobos Cuesta, Luigi Cobuccio, Gianfranco Cocorullo, Elif Colak, Luigi Conti, Yunfeng Cui, Belinda De Simone, Samir Delibegovic, Zaza Demetrashvili, Demetrios Demetriades, Ana Dimova, Agron Dogjani, Mushira Enani, Federica Farina, Francesco Ferrara, Domitilla Foghetti, Tommaso Fontana, Gustavo P. Fraga, Mahir Gachabayov, Grelpois Gérard, Wagih Ghnnam, Teresa Giménez Maurel, Georgios Gkiokas, Carlos A. Gomes, Ali Guner, Sanjay Gupta, Andreas Hecker, Elcio S. Hirano, Adrien Hodonou, Martin Hutan, Igor Ilaschuk, Orestis Ioannidis, Arda Isik, Georgy Ivakhov, Sumita Jain, Mantas Jokubauskas, Aleksandar Karamarkovic, Robin Kaushik, Jakub Kenig, Vladimir Khokha, Denis Khokha, Jae Il Kim, Victor Kong, Dimitris Korkolis, Vitor F. Kruger, Ashok Kshirsagar, Romeo Lages Simões, Andrea Lanaia, Konstantinos Lasithiotakis, Pedro Leão, Miguel León Arellano, Holger Listle, Andrey Litvin, Aintzane Lizarazu Pérez, Eudaldo Lopez-Tomassetti Fernandez, Eftychios Lostoridis, Davide Luppi, Gustavo M. Machain V, Piotr Major, Dimitrios Manatakis, Marianne Marchini Reitz, Athanasios Marinis, Daniele Marrelli, Aleix Martínez-Pérez, Sanjay Marwah, Michael McFarlane, Mirza Mesic, Cristian Mesina, Nickos Michalopoulos, Evangelos Misiakos, Felipe Gonçalves Moreira, Ouadii Mouaqit, Ali Muhtaroglu, Noel Naidoo, Ionut Negoi, Zane Nikitina, Ioannis Nikolopoulos, Gabriela-Elisa Nita, Savino Occhionorelli, Iyiade Olaoye, Carlos A. Ordoñez, Zeynep Ozkan, Ajay Pal, Gian M. Palini, Kyriaki Papageorgiou, Dimitris Papagoras, Francesco Pata, Michał Pędziwiatr, Jorge Pereira, Gerson A. Pereira Junior, Gennaro Perrone, Tadeja Pintar, Magdalena Pisarska, Oleksandr Plehutsa, Mauro Podda, Gaetano Poillucci, Martha Quiodettis, Tuba Rahim, Daniel Rios-Cruz, Gabriel Rodrigues, Dmytry Rozov, Boris Sakakushev, Ibrahima Sall, Alexander Sazhin, Miguel Semião, Taanya Sharda, Vishal Shelat, Giovanni Sinibaldi, Dmitrijs Skicko, Matej Skrovina, Dimitrios Stamatiou, Marco Stella, Marcin Strzałka, Ruslan Sydorchuk, Ricardo A. Teixeira Gonsaga, Joel Noutakdie Tochie, Gia Tomadze, Lara Ugoletti, Jan Ulrych, Toomas Ümarik, Mustafa Y. Uzunoglu, Alin Vasilescu, Osborne Vaz, Andras Vereczkei, Nutu Vlad, Maciej Walędziak, Ali I. Yahya, Omer Yalkin, Tonguç U. Yilmaz, Ali Ekrem Ünal, Kuo-Ching Yuan, Sanoop K. Zachariah, Justas Žilinskas, Maurizio Zizzo, Vittoria Pattonieri, Gian Luca Baiocchi, Fausto Catena

**Affiliations:** 1 Department of Surgery, Macerata Hospital, Macerata, Italy; 2 Department of Surgery, College of Medicine and Health Sciences, UAE University, Al-Ain, United Arab Emirates; 3 Global Alliance for Infections in Surgery, Porto, Portugal; 4 Department of General Surgery, Rambam Health Care Campus, Haifa, Israel; 5 Department of Emergency Surgery, Bufalini Hospital, Cesena, Italy; 6 Abdominal Center, Department of Abdominal Surgery, Helsinki University Hospital Meilahti and University of Helsinki, Helsinki, Finland; 7 General, Acute Care, Abdominal Wall Reconstruction, and Trauma Surgery, Foothills Medical Centre, Calgary, AB Canada; 8 Department of Digestive Surgery and SSPC Research Unit, CHU Amiens-Picardie, Amiens, France; 9 Department of Trauma ICU, IALCH, University of KwaZulu-Natal, Durban, South Africa; 10 Department of General Surgery, University Hospital of Coventry & Warwickshire, Coventry, UK; 11 Department of Surgery, Mansoura University and Emergency Hospital, Mansoura, Egypt; 12 Department of Gastrointestinal Surgery, University of Health Sciences, Elazig Training and Research Hospital, Elazig, Turkey; 13 Department of Surgery, College of Health Sciences, Obafemi Awolowo University, Ile-Ife, Nigeria; 14 Department of Surgery, LAUTECH Teaching Hospital, Osogbo, Nigeria; 15 Department of Surgery, TSMU First University Clinic, Tbilisi, Georgia; 16 Department of General Surgery, Sakarya University Research and Educational Hospital, Sakarya, Turkey; 17 Department of General Surgery, Hacettepe University Hospital, Ankara, Turkey; 18 Department of Surgical Oncology, King Fahad Medical City, Riyadh, Saudi Arabia; 19 Department of General Surgery, Istinye University Faculty of Medicine, Istanbul, Turkey; 20 Department of Primary Care, Primary Health Care Centre of Kissamos, Chania, Greece; 21 Surgical Department, UMHAT “Eurohospital”, Medical University, Plovdiv, Bulgaria; 22 Department of Surgery, University Hospital Centre Zagreb, Zagreb, Croatia; 23 Cirurgia Geral, Centro Hospitalar Universitário da Cova da Beira, Covilhã, Portugal; 24 Department of General Surgery, Hadassah Medical Center, Jerusalem, Israel; 25 Department of Surgery, Saint Savvas Anticancer Hospital, Athens, Greece; 26 General Surgery, Habib bougatfa, Bizerte, Tunisia; 27 Department of Surgery, Lumbini Medical College and Teaching Hospital Ltd., Tansen, Palpa Nepal; 28 Department of Surgery, Hospital San Juan de Dios de La Serena, La Serena, Chile; 29 Emergency and General Surgery, SG Bosco, Torino, Italy; 30 Surgical Department and ICU Department, General Hospital of Larissa, Larissa, Greece; 31 General Surgery, Hospital Santo Tomas, Panama, Panama; 32 Department of Surgery, Elias Emergency Hospital, Bucharest, Romania; 33 Dipartimento Emergenza e Accettazione, Policlinico Umberto I, Roma, Italy; 34 Department of General and Emergency Surgery, ASST Monza - Ospedale San Gerardo, Monza, Italy; 35 General Surgery, South Warwickshire NHS Foundation Trust, Warwick, UK; 36 Department of General Surgery, Kuala Krai Hospital, Kuala Krai, Malaysia; 37 U.O. Chirurgia d’Urgenza Universitaria, Azienda Ospedaliero-Universitaria Pisana, Pisa, Italy; 38 U.O.C. Chirurgia Generale, PO Santissima Trinità, Cagliari, Italia; 39 UGC Cirugía General, Complejo Hospitalario de Jaén, Jaén, Spain; 40 Department of General and Emergency Surgery, Azienda Ospedaliera Policlinico Universitario Palermo “Paolo Giaccone”, Palermo, Italy; 41 General Surgery, University of Health Sciences, Samsun Training and Research Hospital, Samsun, Turkey; 42 Department of Surgery, G. Da Saliceto Hospital, Piacenza, Italy; 43 Department of Surgery, Tianjin Nankai Hospital, Tianjin, China; 44 Chirurgie Viscerale et d’Urgence, Centre Hospitalier Regional de Perpignan, Perpignan, France; 45 Department of Surgery, University Clinical Center Tuzla, Tuzla, Bosnia and Herzegovina; 46 Department of Surgery, Kipshidze Central University Hospital, Tbilisi, Georgia; 47 Division of Trauma and Acute Care Surgery, LAC+USC Medical Center, Los Angeles, USA; 48 Department of General Surgery, University Hospital of Trauma, Tirana, Albania; 49 Department of Infectious Diseases, King Fahad Medical City, Riyadh, Saudi Arabia; 50 Chirurgia Generale, Ospedale Versilia, La Spezia, Italy; 51 Department of Surgery, San Carlo Borromeo Hospital, Milan, Italy; 52 Department of General Surgery, San Salvatore, Pesaro, Italy; 53 Division of Trauma Surgery, Hospital de Clinicas, University of Campinas, Campinas, Brazil; 54 Department of Abdominal Surgery, Vladimir City Clinical Hospital of Emergency Medicine, Vladimir, Russia; 55 Department of Surgery, University hospital, Amiens, France; 56 Department of General Surgery, Mansoura University Hospital, Mansoura, Egypt; 57 Department of General Surgery, Miguel Servet, Zaragoza, Spain; 58 2nd Department of Surgery, Aretaieion University Hospital, National and Kapodistrian University of Athens, Athens, Greece; 59 Department of Surgery, Hospital Universitário Terezinha de Jesus, Faculdade de Ciências Médicas e da Saúde de Juiz de Fora (SUPREMA), Juiz de Fora, Brazil; 60 Department of General Surgery, Karadeniz Technical University, Trabzon, Turkey; 61 Department of General Surgery, Government Medical College and Hospital, Chandigarh, India; 62 Department of General and Thoracic Surgery, University Hospital of Giessen, Giessen, Germany; 63 Department of General Surgery, University and Regional Hospital Center of Borgou, Parakou, Republic of Benin; 64 Chirurgische Abteilung, Landesklinikum Hainburg, Hainburg an der Donau, Austria; 65 Intensive Care Unit, Chernivtsi City Emergency Hospital, Chernivtsi, Ukraine; 66 4th Surgical Department, Medical School, Aristotle University of Thessaloniki, General Hospital “G. Papanikolaou”, Thessaloniki, Greece; 67 Department of General Surgery, Erzincan University Hospital, Erzincan, Turkey; 68 Department of Faculty Surgery #1, Pirogov Russian National Research Medical University, Moscow, Russia; 69 Department of Surgery, SMS Hospital, Jaipur, India; 70 Department of Surgery, Hospital of Lithuanian University of Health Sciences Kaunas Clinics, Kaunas, Lithuania; 71 Faculty of Medicine University of Belgrade Clinic for Surgery, University Clinical Center “Zvezdara”, Belgrade, Serbia; 72 Department of General, Oncologic and Geriatric Surgery, Jagiellonian University Collegium Medicum, Kraków, Poland; 73 Department of Emergency Surgery, City Hospital, Mozyr, Belarus; 74 Department of Vascular Surgery, City Hospital, Mozyr, Belarus; 75 Department of Surgery, Inje University Ilsan Paik Hospital, Goyang, Republic of Korea; 76 Trauma and Acute Care Surgery, Edendale Hospital, Pietermaritzburg, South Africa; 77 Department of Surgery, Krishna Hospital and Medical Research University Karad, Karad, India; 78 Departament of General Surgery, Hospital Municipal de Governador Valadares, Vale do Rio Doce University, Governador Valadares, Brazil; 79 Chirurgia d’Urgenza, Arcispedale Santa Maria Nuova IRCCS, Reggio Emilia, Italy; 80 General Surgery, Scarborough Hospital, York Teaching Hospital NHS FT, York, UK; 81 Cirurgia Geral, Hospital de Braga, Life and Health Sciences Research Institute, ICVS/3Bs, Universidade do Minho, Braga, Portugal; 82 General and Digestive Surgery, Hospital Fundación Jimenez Diaz, Madrid, Spain; 83 General, Visceral, Thoracic and Vascular Surgery, University Hospital Greifswald, Greifswald, Germany; 84 Department of Surgical Disciplines, Regional Clinical Hospital, Immanuel Kant Baltic Federal University, Kaliningrad, Russia; 85 Cirugía general y del aparato digestivo, Hospital Universitario Donostia, Donostia, Spain; 86 Gastrointestinal Surgery, Hospital Insular de Gran Canaria, Las Palmas de Gran Canaria, Spain; 87 1st Department of Surgery, Kavala General Hospital, Kavala, Greece; 88 Department of General and Emergency Surgery, ASMN Reggio Emilia, Modena, Italy; 89 II Catedra de Clinica Quirúrgica, Hospital de Clinicas, Facultad de Ciencias Médicas, Universidad Nacional de Asunción, Asunción, Paraguay; 90 2nd Department of General Surgery, Jagiellonian University Medical College, Kraków, Poland; 91 Department of Surgery, Athens Naval and Veterans Hospital, Athens, Greece; 92 First Department of Surgery, Tzaneio General Hospital, Piraeus, Greece; 93 Department of General Surgery and Surgical Oncology, Policlinico Le Scotte, University of Siena, Siena, Italy; 94 Department of General and Digestive Surgery, Hospital Universitario Doctor Peset, Valencia, Spain; 95 Department of General Surgery, Post-graduate Institute of Medical Sciences, Rohtak, India; 96 Department of Surgery, Radiology, Anaesthesia and Intensive Care, University Hospital of the West Indies, Kingston, Jamaica; 97 Second Surgical Clinic, Emergency County Hospital of Craiova, Craiova, Romania; 98 3rd Department of Surgery, Ahepa University Hospital, Thessaloniki, Greece; 99 3rd Department of Surgery, Attikon University Hospital, Athens, Greece; 100 Department of Surgery, Hassan II, Fez, Morocco; 101 Department of Specialist Surgery, Port Shepstone Regional Hospital, Port Shepstone, Republic of South Africa; 102 General Surgery Department, Emergency Hospital of Bucharest, Bucharest, Romania; 103 Toxicology and Sepsis, Riga East University Hospital, Riga, Latvia; 104 Department of General Surgery, Queen Elizabeth Hospital, London, UK; 105 Chirurgia generale, Sant’Anna (AUSL Reggio Emilia), Castelnovo ne’ Monti, Italy; 106 U.O. Chirurgia d’Urgenza, Arcispedale S. Anna Ferrara, Ferrara, Italy; 107 Department of Surgery, University of Ilorin Teaching Hospital, Ilorin, Nigeria; 108 Department of Surgery, Fundacion Valle del Lili - Universidad del Valle, Cali, Colombia; 109 Department of General Surgery, University of Health Sciences, Elazig Training and Research Hospital, Elazig, Turkey; 110 Department of Surgery, King George’s Medical University, Lucknow, India; 111 Chirurgia Generale e d’Urgenza, Ospedale Infermi, Rimini, Italy; 112 Surgical Oncology, University Hospital Heraclion Crete, Heraclion Crete, Greece; 113 Department of General Surgery, General Hospital of Trikala, Trikala, Greece; 114 Department of Surgery, Sant’Antonio Abate Hospital, Gallarate, Italy; 115 Department of General and Emergency Surgery, University Hospital, University Hospital Kraków, Kraków, Poland; 116 Cirurgia Geral, Centro Hospitalar Tondela-Viseu, Viseu, Portugal; 117 Medicina, Base Hospital, Bauru, Brazil; 118 Chirurgia d’Urgenza – Dipartimento Urgenza/Emergenza, AOU Parma, Parma, Italy; 119 Department of Abdominal Surgery, UMC Ljubljana, Ljubljana, Slovenia; 120 Department of Endoscopic, Metabolic and Soft Tissue Tumors Surgery, University Hospital, Kraków, Poland; 121 Surgery Department, Chernivtsi City Emergency Hospital, Chernivtsi, Ukraine; 122 Department of General, Emergency and Robotic Surgery, San Francesco Hospital, Nuoro, Italy; 123 Department of Surgery, AO San Giovanni Addolorata, Rome, Italy; 124 Department of Surgery/Trauma, Hospital Santo Tomás, Panama, Panama; 125 Department of Gastrointestinal Surgery, HGR1 IMSS, Cuernavaca, Mexico; 126 Department of General Surgery, Kasturba Medical College, Manipal Academy of Higher Education, Manipal, India; 127 First Clinic of General Surgery, University Hospital St George/Medical University Plovdiv, Plovdiv, Bulgaria; 128 Chirurgie Générale et Viscérale, Hôpital d’instruction des Armées, Hôpital Principal de Dakar, Dakar, Senegal; 129 Department of General Surgery, Tan Tock Seng Hospital, Singapore, Singapore; 130 Department of Surgery, Fatebbenefratelli Hospital, Isola Tiberina, Rome, Italy; 131 Department of Surgery (Department No. 10), Riga East Clinical University Hospital “Gaiļezers”, Riga, Latvia; 132 Department of Surgery, Hospital and Oncological Centre Novy Jicin, Novy Jicin, Czech Republic; 133 General Surgery, Heartlands Hospital, Birmingham, UK; 134 Department of General Surgery, Polytrauma and Emergency Medicine, University Hospital of the Jagiellonian University Medical College, Kraków, Poland; 135 General Surgery Department, Bukovinian State Medical University, Chernivtsi, Ukraine; 136 Trauma and Emergency Surgery, Hospital Escola Padre Albino, Catanduva, Brazil; 137 Faculty of Medicine and Biomedical Sciences, University of Yaounde I, Yaounde, Cameroon and Department of Surgery and Anaesthesiology, Yaounde Central Hospital, Yaounde, Cameroon; 138 Surgery Department, Tbilisi State Medical University, Tbilisi, Georgia; 139 Chirurgia Generale, Ospedale Civile di Guastalla, Reggio Emilia, Italy; 140 First Department of Surgery, Department of Abdominal, Thoracic Surgery and Traumatology, First Faculty of Medicine, Charles University and General University Hospital, Prague, Czech Republic; 141 Upper Gastrointestinal Tract Surgery Department, North Estonia Medical Centre, Tallinn, Estonia; 142 Department of General Surgery, Siirt State Hospital, Siirt, Turkey; 143 First Surgical Unit, “St. Spiridon” University Hospital Iasi, University of Medicine and Pharmacy “Grigore T. Popa”, Iasi, Romania; 144 Renal Transplant and General Surgery, Manchester Royal Infirmary, Manchester, UK; 145 Department of Surgery, Clinical Center University of Pecs, Pecs, Hungary; 146 Department of General, Oncological, Metabolic and Thoracic Surgery, Military Institute of Medicine, Warsaw, Poland; 147 Department of Surgey, Zliten Teaching Hospital, Zliten, Libya; 148 Department of General Surgery, Ankara University School of Medicine, Ankara, Turkey; 149 Transplantation Unıt, Acibadem Atakent Hospital, İstanbul, Turkey; 150 Department of Surgery, Taipei Medical University Hospital, Taipei, Taiwan; 151 Department of Surgery, Mosc Medical College, Kolenchery, Cochin, India; 152 Surgical Oncology Unit, Azienda Unità Sanitaria Locale - IRCCS di Reggio Emilia, Reggio Emilia, Italy; 153 Emergency Surgery Department, Maggiore Parma Hospital, Parma, Italy; 154 Department of Clinical and Experimental Sciences, University of Brescia, Brescia, Italy

**Keywords:** Acute peritonitis, Source control, Early warning score, Emergency surgery

## Abstract

**Background:**

Timing and adequacy of peritoneal source control are the most important pillars in the management of patients with acute peritonitis. Therefore, early prognostic evaluation of acute peritonitis is paramount to assess the severity and establish a prompt and appropriate treatment. The objectives of this study were to identify clinical and laboratory predictors for in-hospital mortality in patients with acute peritonitis and to develop a warning score system, based on easily recognizable and assessable variables, globally accepted.

**Methods:**

This worldwide multicentre observational study included 153 surgical departments across 56 countries over a 4-month study period between February 1, 2018, and May 31, 2018.

**Results:**

A total of 3137 patients were included, with 1815 (57.9%) men and 1322 (42.1%) women, with a median age of 47 years (interquartile range [IQR] 28–66). The overall in-hospital mortality rate was 8.9%, with a median length of stay of 6 days (IQR 4–10). Using multivariable logistic regression, independent variables associated with in-hospital mortality were identified: age > 80 years, malignancy, severe cardiovascular disease, severe chronic kidney disease, respiratory rate ≥ 22 breaths/min, systolic blood pressure < 100 mmHg, AVPU responsiveness scale (voice and unresponsive), blood oxygen saturation level (SpO_2_) < 90% in air, platelet count < 50,000 cells/mm3, and lactate > 4 mmol/l. These variables were used to create the PIPAS Severity Score, a bedside early warning score for patients with acute peritonitis. The overall mortality was 2.9% for patients who had scores of 0–1, 22.7% for those who had scores of 2–3, 46.8% for those who had scores of 4–5, and 86.7% for those who have scores of 7–8.

**Conclusions:**

The simple PIPAS Severity Score can be used on a global level and can help clinicians to identify patients at high risk for treatment failure and mortality.

## Introduction

Peritonitis is an inflammation of the peritoneum. Depending on the underlying pathology, it can be infectious or sterile [1]. Infectious peritonitis is classified into primary peritonitis, secondary peritonitis, and tertiary peritonitis. Primary peritonitis is a diffuse bacterial infection (usually caused by a single organism) without loss of integrity of the gastrointestinal tract, typically seen in cirrhotic patients with ascites or in patients with a peritoneal dialysis catheter. It has a low incidence in surgical wards and is usually managed without any surgical intervention. Secondary peritonitis is an acute peritoneal infection resulting from loss of integrity of the gastrointestinal tract. Tertiary peritonitis is a recurrent infection of the peritoneal cavity that occurs > 48 h after apparently successful and adequate surgical source control of secondary peritonitis. Secondary peritonitis is the most common form of peritonitis. It is caused by perforation of the gastrointestinal tract (e.g. perforated duodenal ulcer) by direct invasion from infected intra-abdominal viscera (e.g. gangrenous appendicitis). It is an important cause of patient morbidity and is frequently associated with significant morbidity and mortality rates [2], despite development in diagnosis and management.

Timing and adequacy of peritoneal source control are the most important pillars in the management of patients with acute peritonitis, being determinant to control or interrupt the septic process [2, 3].

Many peritonitis-specific scoring systems have been designed and used to grade the severity of acute peritonitis [4–7].

Patients with acute peritonitis are generally classified into low risk and high risk. “High risk” is generally intended to describe patients at high risk for treatment failure and mortality [6]. In high-risk patients, the increased mortality associated with inappropriate management cannot be reversed by subsequent modifications. Therefore, early prognostic evaluation of acute peritonitis is important to assess the severity and decide the aggressiveness of treatment. Moreover, in emergency departments of limited-resource hospitals, diagnosis of acute peritonitis is mainly clinical, and supported only by basic laboratory tests [8], making some scoring systems impractical to a large part of the world’s population.

The objectives of this study were (a) to identify all clinical and laboratory predictors for in-hospital mortality in patients with acute peritonitis and (b) to develop a warning score system, based on easily recognizable and assessable variables, globally accepted, so as to provide the clinician with a simple tool to identify patients at high risk for treatment failure and mortality.

## Methods

### Study population

This worldwide multicentre observational study was performed across 153 surgical departments from 56 countries over a 4-month study period (February 1, 2018 – May 31, 2018). All consecutive patients admitted to surgical departments with a clinical diagnosis of acute peritonitis were included in the study. The following data were collected: age and gender; presence of comorbidities, namely primary or secondary immunodeficiency (chronic treatment with glucocorticoids, with immunosuppressive agents or chemotherapy, and patients with lymphatic diseases or with virus-related immunosuppression; solid or haematopoietic and lymphoid malignancy; severe cardiovascular disease (medical history of ischemic heart disease, history of heart failure, severe valvular disease [9]); diabetes with or without organ dysfunction; severe chronic kidney disease; and severe chronic obstructive pulmonary disease (COPD) [10]. Clinical findings were recorded at admission: abdominal findings (localized or diffuse abdominal pain, localized or diffuse abdominal rigidity); core temperature (defining fever as core temperature > 38.0 °C, and hypothermia as core temperature < 36.0 °C); heart rate (bpm); respiratory rate (breaths/min); systolic blood pressure (mmHg); alert/verbal/painful/unresponsive (AVPU) responsiveness scale [11]; and numerical rating scale (NRS) [12].

The following laboratory findings were also collected: blood oxygen saturation level (SpO_2_) (%) in air, white blood count (WBC) (cells/mm^3^), platelet count (cells/ mm^3^), international normalised ratio (INR), C-reactive protein (CRP) (mg/l), procalcitonin (ng/ml), and lactate (mmol/l). Quick Sequential Organ Failure Assessment (qSOFA) score upon admission was calculated [13]. The modality and setting of acquisition of radiological investigations (abdominal x-ray, ultrasound [US], computer tomography [CT] scan) was specified. Peritonitis was classified as community-acquired or healthcare-acquired. Peritonitis was considered healthcare-associated in patients hospitalized for at least 48 h during the previous 90 days; or those residing in skilled nursing or long-term care facility during the previous 30 days; or those who have received intravenous therapy, wound care, or renal replacement therapy within the preceding 30 days. Source of infection, extent of peritonitis (generalized or localized peritonitis/abscess), source control (conservative treatment, operative or non-operative interventional procedures), and its adequacy were noted. The adequacy of the intervention was defined by the establishment of the cause of peritonitis and the ability to control the source of the peritonitis [14]. Delay in the initial intervention (> 24 h of admission), and adequacy of antimicrobial therapy (if guided by antibiograms performed) were assessed. Reoperation during the hospital stay, re-laparotomy strategy (open abdomen, planned re-laparotomy, on demand re-laparotomy) and its timing, immediate (within 72 h) infectious post-operative complications, delayed infectious post-operative complications, length of hospital stay (LOS), and in-hospital mortality were determined. All patients were monitored until they were discharged or transferred to another facility.

### Study design

The centre coordinator of each participating medical institution collected data in an online case report database. Differences in local surgical practice of each centre were respected, and no changes were impinged on local management strategies. Each centre followed its own ethical standards and local rules. The study was monitored by a coordinating centre, which processed and verified any missing or unclear data submitted to the central database. The study did not attempt to change or modify the clinical practice of the participating physicians. Accordingly, informed consent was not needed and each hospital followed their ethical rules for formal research including an ethical approval if approval was needed. The data were completely anonymised. The study protocol was approved by the board of the World Society of Emergency Surgery (WSES), and the study was conducted under its supervision. The board of the WSES granted the proper ethical conduct of the study. The study met and conformed to the standards outlined in the Declaration of Helsinki and Good Epidemiological Practices.

### Statistical analysis

The data were analysed in absolute frequency and percentage, in the case of qualitative variables. Quantitative variables were analysed as medians and interquartile range (IQR). Univariate analyses were performed to study the association between risk factors and in-hospital mortality using a chi-square test, or a Fisher’s exact test, if the expected value of a cell was < 5. All tests were two-sided, and p values of 0.05 were considered statistically significant.

To identify independent risk factors associated with in-hospital mortality, a multivariable logistic regression analysis was performed selecting independent variables that had *p* value < 0.05 in the univariate analysis. Then, a backward selection method was applied to select a limited number of variables, using a likelihood ratio test for comparing the nested models (*α* = 0.05). At each step, we removed from the previous model the variable with the highest *p* value greater than α, checking the fit of the obtained model, and then stopping when all *p* values were less than α. Then, we checked the global performance of the test calculating the area under the receiver operating characteristic (ROC) curve. All statistical analyses were performed using the Stata 11 software package (StataCorp, College Station, TX).

## Results

### Patients and diagnosis

During the study, 3137 patients from 153 hospitals worldwide were collected; these included 1815 (57.9%) men and 1322 (42.1%) women, with a median age of 47 years (IQR, 28–66). Considering World Health Organization regions, 1981 (63.1%) patients were collected in countries belonging to European region, 396 (12.6%) patients were from the African region, 275 (8.8%) from the region of the Americas, 239 (7.6%) from the South-East Asia region, 173 (5.5%) from the Eastern-Mediterranean region, and 73 (2.3%) from the Western Pacific region.

Forty-one (1.3%) patients were asymptomatic, while 990 (31.6%) reported localized abdominal pain, 665 (21.2%) localized abdominal rigidity, 797 (25.4%) diffuse abdominal pain, and 592 (18.9%) diffuse abdominal rigidity. In 52 (1.7%) patients, abdominal findings were not reported. Three hundred and thirty (10.5%) patients underwent abdominal x-ray, 756 (24.1%) patients had an US, 1016 (32.4%) abdominal CT scan, 189 (6.0%) patients had both abdominal x-ray and US, 76 (2.4%) had both abdominal x-ray scan and CT, 199 (6.3%) patients had both CT scan and US, 93 (3.0%) patients underwent abdominal x-ray scan, US and CT, and 445 (14.3%) patient did not undergo any radiological investigation. In 33 (1.1%) patients, radiological diagnosis was not specified.

Considering the setting of acquisition, 2826 (90.1%) patients were affected by community-acquired intra-abdominal infections (IAIs), while the remaining 311 (9.9%) suffered from healthcare-associated IAIs; moreover, 1242 patients (39.6%) were affected by generalized peritonitis, while 1895 (60.4%) suffered from localized peritonitis or abscesses. The cause of infection was acute appendicitis in 1321 (42.1%) patients, acute cholecystitis in 415 (13.2%), gastroduodenal perforation in 364 (11.6%) patients, small bowel perforation in 219 (7.0%), acute diverticulitis in 217 (6.9%), colonic perforation in 203 (6.5%), post-traumatic perforation in 79 (2.5%), acute infected pancreatitis in 40 (1.3%), pelvic inflammatory disease (PID) in 30 (1.0%), and other causes in 249 (7.9%).

### Management

Among all patients enrolled in the PIPAS Study, 377 (12%) underwent non-operative procedures, and the other 2760 (88.0%) patients underwent operative interventional procedures as first-line treatment. Source control was considered inadequate in 247 (247/2834, 8.7%) patients who underwent surgical procedures. In 1630 (1630/2834, 57.5%) patients the initial intervention was delayed. Among 2159 patients who received antimicrobial therapy, in 336 (15.6%), it was considered inadequate. During the same hospitalization, 242 (242/2760, 8.8%) patients underwent a second procedure after 4 (IQR 2–7) days because of a postoperative complication or a worsening of the initial stage. In particular, 79 (2.9%) patients underwent an open abdomen surgery, 57 (2.1%) a planned relaparotomy, and 87 (3.2%) an on-demand relaparotomy, and in 19 (0.7%) patients, no specific procedure was specified.

Immediate post-operative complications were observed in 339 (339/2760, 12.3%) patients who underwent a surgical procedure; among them we observed ongoing peritonitis in 174 (6.3%) patients, multi-organ failure in 33 (1.2%), bleeding in 32 (1.2%), cardiovascular complications in 17 (0.6%), respiratory complications in 15 (0.5%), sepsis or septic shock in 13 (0.5%), and other complications in 55 (2.0%). Delayed post-operative complications were detected in 774 (774/2760, 28.0%) patients who underwent an interventional procedure; in particular, they suffered from surgical site infections in 343 (12.4%) patients, post-operative peritonitis in 132 (4.8%), post-operative abdominal abscess in 118 (4.3%), respiratory complications in 54 (2.0%),cardiovascular complications in 39 (1.4%), sepsis or septic shock in 33 (1.2%), ileus in 22 (0.8%), multi-organ failure in 18 (0.7%), renal complications in 13 (0.5%), and other complications in 79 (2.9%).

### Outcome

The overall in-hospital mortality rate was 8.9%. The median duration of hospitalization was 6 days (IQR 4–10). Bivariate analyses were performed to analyse the association between risk factors and in-hospital mortality using a two-sided chi-square test or a two-sided Fisher’s exact test where appropriate. Distribution of clinical predictive variables of in-hospital mortality is reported in Table 1. Distribution of laboratory predictive variables of in-hospital mortality is reported in Table 2.

**Table 1:** Distribution of clinical predictive variables of in-hospital mortality

Variables	Total patients	Dead	Survivors	RR	*p* value
	*n* 3137	*n* 280	*n* 2857		
	(100%)	(8.9%)	(91.1%)		
Age > 80 years	246 (7.8)	72 (25.7)	174 (6.1)	4.07 (3.22–5.14)	< 0.001
Immunodeficiency	240 (7.7)	56 (20.0)	184 (6.4)	3.02 (2.32–3.92)	< 0.001
Malignancy	333 (10.6)	83 (29.6)	250 (8.8)	3.55 (2.82–4.46)	< 0.001
Severe cardiovascular disease	406 (12.9)	106 (37.9)	300 (10.5)	4.10 (3.30–5.10)	< 0.001
Diabetes	400 (12.8)	76 (27.1)	324 (11.3)	2.55 (2.00–3.25)	< 0.001
Severe CKD	141 (4.5)	52 (18.6)	89 (3.1)	4.85 (3.78–6.22)	< 0.001
Severe COPD	186 (5.9)	60 (21.4)	126 (4.4)	4.33 (3.39–5.52)	< 0.001
Core temperature (°C)					
< 36.0	85 (2.7)	23 (8.2)	62 (2.2)	3.21 (2.22–4.64)	< 0.001
36.0–38.0	2292 (73.1)	185 (66.1)	2107 (73.7)	0.72 (0.57–0.91)	< 0.05
> 38.0	760 (24.2)	72 (25.7)	688 (24.1)	1.08 (0.84–1.40)	0.54
Hearth rate (bpm)					
< 60	8 (0.3)	1 (0.4)	7 (0.2)	1.40 (0.22–8.80)	0.72
60–100	1919 (61.2)	117 (41.8)	1802 (63.1)	0.46 (0.36–0.57)	< 0.001
> 100	1210 (38.6)	162 (57.9)	1048 (36.7)	2.19 (1.74–2.74)	< 0.001
Systolic blood pressure (mmHg)					
< 90	138 (4.4)	49 (17.5)	89 (3.1)	4.61 (3.57–5.96)	< 0.001
90–100	388 (12.4)	70 (25.0)	318 (11.1)	2.36 (1.84–3.03)	< 0.001
> 100	2610 (83.2)	161 (57.5)	2449 (85.7)	0.27 (0.22–0.34)	< 0.001
Respiratory rate (breaths/min)					
< 22	2244 (71.5)	124 (44.3)	2120 (74.2)	0.32 (0.25–0.40)	< 0.001
22–29	684 (21.8)	97 (34.6)	587 (20.5)	1.90 (1.50–2.39)	< 0.001
30–35	154 (4.9)	39 (13.9)	115 (4.0)	3.13 (2.33–4.21)	< 0.001
> 35	55 (1.8)	20 (7.1)	35 (1.2)	4.31 (2.98–6.23)	< 0.001
AVPU responsiveness scale					
Alert	2917 (93.0)	187 (66.8)	2730 (95.6)	0.15 (0.12–0.19)	< 0.001
Voice	123 (3.9)	54 (19.3)	69 (2.4)	5.85 (4.62–7.41)	< 0.001
Pain	74 (2.4)	23 (8.2)	51 (1.8)	3.70 (2.59–5.30)	< 0.001
Unresponsive	23 (0.7)	16 (5.7)	7 (0.2)	8.21 (6.12–11.01)	< 0.001
NRS					
0–3	80 (2.6)	16 (5.7)	64 (2.2)	2.32 (1.47–3.64)	< 0.001
4–6	1512 (48.2)	112 (40.0)	1400 (49.0)	0.72 (0.57–0.90)	< 0.05
7–10	1112 (35.4)	128 (45.7)	984 (34.4)	1.53 (1.23–1.92)	< 0.001
Not reported	433 (13.8)	24 (8.6)	409 (14.3)	NA	NA
qSOFA score					
0	1367 (43.6)	37 (13.2)	1330 (46.6)	0.20 (0.14–0.28)	< 0.001
1	1323 (42.2)	109 (38.9)	1214 (42.5)	0.87 (0.96–1.10)	0.25
2	353 (11.3)	84 (30.0)	269 (9.4)	3.38 (2.68–4.26)	< 0.001
3	94 (3.0)	50 (17.9)	44 (1.5)	7.04 (5.61–8.82)	< 0.001

All *p* values calculated using two-sided chi-square test

*RR*: risk ratio, *NA*: not applicable, *CKD*: chronic kidney disease, *COPD*: chronic obstructive pulmonary disease, *AVPU*: alert/verbal/painful/unresponsive, *NRS*: numerical rating scale, *qSOFA*: Quick Sequential Organ Failure Assessment

**Table 2:** Distribution of laboratory predictive variables of in-hospital mortality

Variables	Total patients	Dead	Survivors	RR	*p* value
	*n* 3137	*n* 280	*n* 2857		
	(100%)	(8.9%)	(91.1%)		
Blood oxygen saturation level (SpO_2_) (%) in air					
> 92	2782 (88.7)	152 (54.3)	2630 (92.1)	0.15 (0.12–0.19)	< 0.001
90–91	198 (6.3)	66 (23.6)	132 (4.6)	4.58 (3.62–5.79)	< 0.001
85–89	99 (3.1)	41 (14.6)	58 (2.0)	5.26 (4.04–6.85)	< 0.001
< 85	21 (0.7)	9 (3.2)	12 (0.4)	4.93 (2.97–8.18)	< 0.001
Not reported	37 (1.2)	12 (4.3)	25 (0.9)	NA	NA
WBC (cells/mm^3^)					
> 12,000	1950 (62.2)	182 (65.0)	1768 (61.9)	1.13 (0.89–1.43)	0.30
4000–12,000	1043 (33.2)	63 (22.5)	980 (34.3)	0.58 (0.44–0.76)	< 0.001
< 4000	94 (3.0)	29 (10.4)	65 (2.3)	3.74 (2.70–5.18)	< 0.001
Not reported	50 (1.6)	6 (2.1)	44 (1.5)	NA	NA
Platelet count (cells/ mm^3^)					
> 150,000	2606 (83.1)	183 (65.4)	2423 (84.8)	0.38 (0.31–0.49)	< 0.001
50,000–1,500,000	387 (12.3)	73 (26.1)	314 (11.0)	2.51 (1.96–3.20)	< 0.001
< 50,000	32 (1.0)	18 (6.4)	14 (0.5)	6.67 (4.81–9.24)	< 0.001
Not reported	112 (3.6)	6 (2.1)	106 (3.7)	NA	NA
INR					
> 3	23 (0.7)	12 (4.3)	11 (0.4)	6.06 (4.03–9.11)	< 0.001
1.2–3	296 (9.4)	72 (25.7)	224 (7.8)	3.32 (2.61–4.22)	< 0.001
< 1.2	1954 (62.3)	149 (53.2)	1805 (63.2)	0.69 (0.55–0.86)	0.001
Not reported	864 (27.5)	47 (16.8)	817 (28.6)	NA	NA
CRP (mg/l)					
> 200	450 (14.3)	70 (25.0)	380 (13.3)	1.99 (1.55–2.56)	< 0.001
101–200	462 (14.7)	51 (18.2)	411 (14.4)	1.29 (0.97–1.72)	0.08
5–100	946 (30.2)	69 (24.6)	877 (30.7)	0.76 (0.58–0.98)	0.04
< 5	258 (8.2)	3 (1.1)	255 (8.9)	0.12 (0.04–0.37)	< 0.001
Not reported	1471 (46.9)	157 (56.1)	1314 (46.0)	NA	NA
Procalcitonin (ng/ml)					
> 10	85 (2.7)	31 (11.1)	54 (1.9)	4.47 (3.30–6.06)	< 0.001
0.5–10	260 (8.3)	42 (15.0)	218 (7.6)	1.96 (1.44–2.64)	< 0.001
< 0.5	100 (3.2)	3 (1.1)	97 (3.4)	0.33 (0.11–1.01)	0.03
Not reported	2692 (85.8)	204 (72.9)	2488 (87.1)	NA	NA
Lactate (mmol/l)					
>4	139 (4.4)	61 (21.8)	78 (2.7)	6.01 (4.79–7.54)	< 0.001
1–4	615 (19.6)	86 (30.7)	529 (18.5)	1.82 (1.43–2.31)	< 0.001
< 1	136 (4.3)	6 (2.1)	130 (4.6)	0.48 (0.22–1.07)	0.06
Not reported	2247 (71.6)	127 (45.4)	2120 (74.2)	NA	NA

All *p* values calculated using two-sided chi-square test

*RR*: risk ratio, *NA*: not applicable, *WBC*: white blood count, *INR*: international normalised ratio, *CRP*; C-reactive protein

Independent variables associated with in-hospital mortality according to the multivariable logistic regression are reported in Table 3. The model was highly significant (*p* < 0.0001), and the global performance of the test is explained by the area under the ROC curve, which is equals to 0.84 (95% CI).

**Table 3:** Results of multinomial logistic regression for the analysis of variables associated with in-hospital mortality

Variables	OR	95% CI	*p* value
Age > 80 years	2.11	1.43–3.10	< 0.001
Malignancy	3.02	2.15–4.24	< 0.001
Severe cardiovascular disease	2.76	1.97–3.87	< 0.001
Severe chronic kidney disease	3.33	2.12–5.23	< 0.001
Respiratory rate ≥ 22 breaths/min	3.38	2.23–5.13	< 0.001
Systolic blood pressure < 100 mmHg	2.18	1.58–3.00	< 0.001
AVPU responsiveness scale voice or unresponsive	3.07	2.10–4.51	< 0.001
Blood oxygen saturation level (SpO_2_) < 90% in air	2.67	1.64–4.32	< 0.001
Platelet count < 50,000 cells/ mm^3^	4.81	2.07–11.20	< 0.001
Lactate > 4 mmol/l	4.00	2.58–6.23	< 0.001

*CI*: confidence interval, *OR*: odds ratio, *AVPU*: alert/verbal/painful/unresponsive

### Developing the severity score

The second aim of the study was to develop a severity score for patients with a clinical diagnosis of acute peritonitis that is simple and globally acceptable with a good prognostic value. Only the significant clinical variables associated with in-hospital mortality obtained from the multivariable logistic regression model were included, excluding the lactate, and platelet count. This modification was done for three reasons: (a) to simplify the score, (b) to make it more universal and globally acceptable, and (c) because of lack of facilities to obtain lactate in low-income countries. The coefficients of the variables were used to develop the score, and not the Odds Ratio. The significant clinical variables were subjected to different direct logistic regression models using either simple binomial variables or ordinal data, to arrive at a simplified and acceptable model. Direct logistic regression model of the clinical variables affecting mortality which were used to develop the score is reported in Table 4. The score would have become complicated if we had to follow the model proposed by Moons et al. [15], whereby the coefficient would have to be multiplied by 10 and the value approximated to the nearest integral to get a score. This meant that the scores derived from the model would be 10, 11, 9, 12, 8, 9, 9, and 14, making it very complex. Hence, it was decided to approximate the coefficient to the nearest integral number and test the model. Since the coefficients were approximated to 1, each of these variables could have a score of 1 or 0 with a maximum score of 8 and a range of 0–8. The simplified and finalized the PIPAS Severity Score is shown in the Appendix.

**Table 4:** Direct logistic regression model with clinical variables affecting mortality of patients used to develop the score

Variable	Estimate	SE	Wald test	*P*	OR	95% CI	
						LL	UL
Age > 80 years	0.97	0.19	25.91	< 0.0001	2.63	1.81	3.89
Malignancy	1.13	0.17	42.43	< 0.0001	3.11	2.21	4.37
Severe CVD	0.88	0.17	26.09	< 0.0001	2.41	1.72	3.38
Severe CKD	1.2	0.23	26.23	< 0.0001	3.32	2.1	5.26
RR ≥ 22 breaths/min	0.75	0.16	22.61	< 0.0001	2.11	1.55	2.87
SBP < 100 mmHg	0.86	0.17	27.29	< 0.0001	2.37	1.71	3.27
AVPU responsiveness scale: not completely alert.	1.35	0.2	47.98	< 0.0001	3.86	2.63	5.65
Blood oxygen saturation level: SpO_2_ < 90% in air	0.87	0.25	12.15	< 0.0001	2.39	1.46	3.89
Constant	− 3.79	0.13	834.77	< 0.0001	0.023	–	–

*SE*: standard error, *OR*: odds ratio, *CI*: confidence interval, *LL*: lower limit, *UL*: upper limit, *CVD*: cardiovascular disease, *CKD*: chronic kidney disease, *RR*: respiratory rate, *SBP*: systolic blood pressure, *AVPU*: alert/verbal/painful/unresponsive

**Fig. 1:**
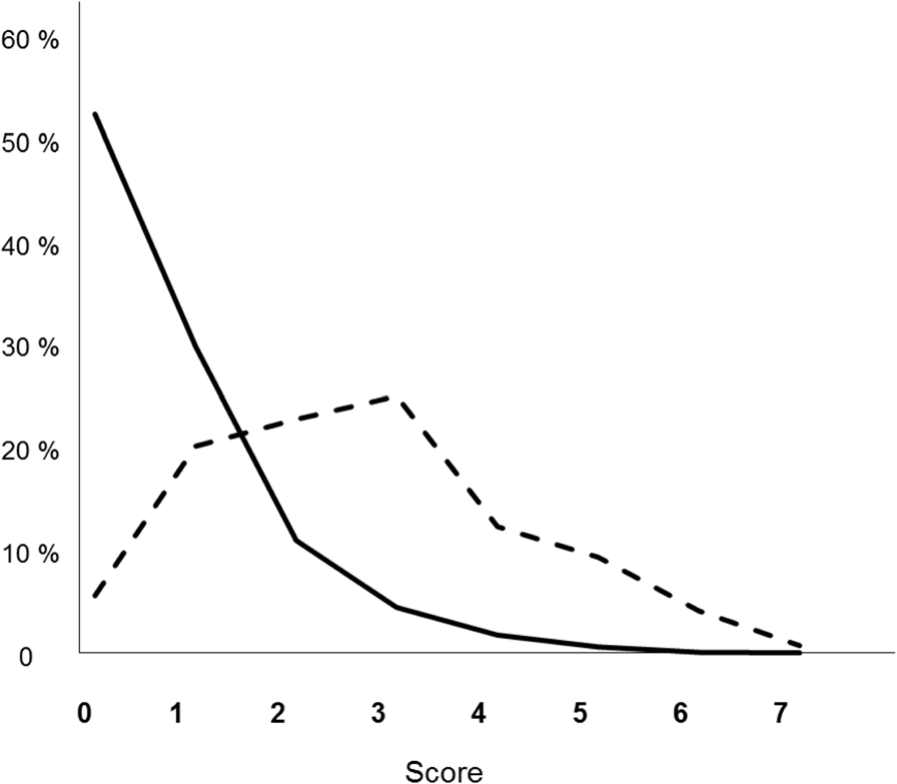
Distribution of the percentile PIPAS Severity Score of hospitalized peritonitis patients for those who survived (*continuous line*) (*n* = 2832) and those who died (*interrupted line*) (*n* = 268). Global data from 153 worldwide surgical departments in 56 countries, over a 4-month study period (February 1, 2018–May 31, 2018). Thirty-seven patients (1.2%) had missing data in whom the score could not be computed

**Fig. 2:**
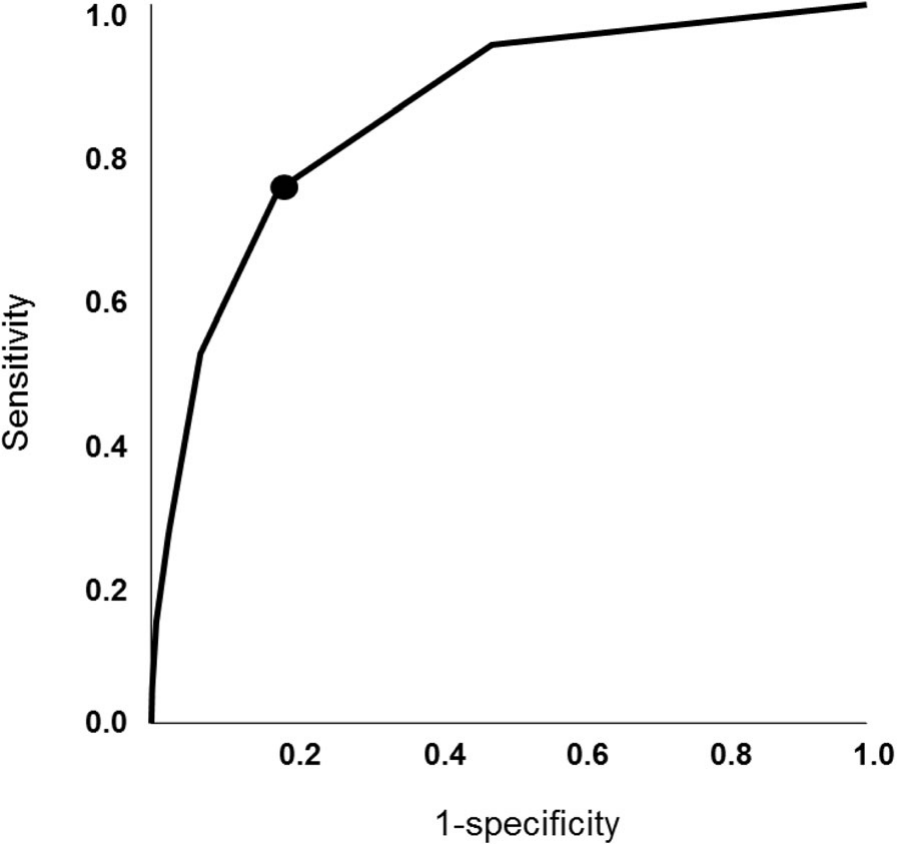
Receiver operating characteristic (ROC) curve for the best PIPAS Severity Score (1.5, black circle) that predicted mortality in peritonitis patients. Global data from 153 worldwide surgical departments in 56 countries, over a 4-month study period (February 1, 2018–May 31, 2018)

The PIPAS Severity Score had a very good ability of distinguishing those who survived from those who died (Fig. 1). The ROC curve showed that the best cutoff point for predicting mortality was a PIPAS Severity Score of 1.5 having a sensitivity of 74.3%, a specificity of 82.2% (Fig. 2) and an area under the curve of 85.1%. The overall mortality was 2.9% for the patients who had scores of 0 and 1, 22.7% for those who had scores of 2 and 3, 46.8% for those who had scores 4 and 5, and 86.7% for those who have scores 7–8.

## Discussion

Using the multivariable logistic regression, ten independent variables associated with in-hospital mortality were identified. The model was highly significant, with a good global performance of the test. Excluding platelet count and lactate, eight bedside easy-to-measure parameters were recognized to develop an early warning score, the PIPAS Severity Score, assessing anamnestic data (age > 80 years, malignancy, severe cardiovascular disease, severe chronic kidney disease), and physiological functions (respiratory rate ≥ 22 breaths/min, systolic blood pressure < 100 mmHg, AVPU responsiveness scale voice or unresponsive, blood oxygen saturation level (SpO_2_) < 90% in air).

The PIPAS Severity Score, taking into account physiological parameters recognizable on hospital admission, immediately allows clinicians to assess the severity and decide the aggressiveness of treatment. Particularly for clinicians working in low- and middle-income countries, where diagnostic imaging is often insufficient, and in some instances completely lacking, the utility of this score system is remarkable [16].

Sometimes, the atypical clinical presentation of acute peritonitis may be responsible for a delay in diagnosis and treatment. Therefore, a triage system that quickly recognizes patients at high risk for mortality and allows to transfer them immediately to an acute care unit is a vital component of the emergency services. As a consequence, any process of improving the quality of emergency care globally should focus on simple diagnostic criteria based on physical examination findings that can recognize patients needing critical care. From a global perspective, a feasible, low-cost method of rapidly identifying patients requiring critical care is crucial. Early warning system scores utilize physiological, easy-to-measure parameters, assessing physiological parameters such as systolic blood pressure, pulse rate, respiratory rate, temperature, oxygen saturations, and level of consciousness [17].

The statistical analysis shows that the PIPAS Severity Score has a very good ability of distinguishing those who survived from those who died. The overall mortality was 2.9% for the patients who had scores of 0 and 1, 22.7% for those who had scores of 2 and 3, 46.8% for those who had scores of 4 and 5, and 86.7% for those who have scores of 7–8.

PIPAS Study has strengths and limitations. It is an observational multicentre study involving a large, but probably not representative, number of hospitals worldwide, since the majority of patients were collected in countries belonging to the WHO European region. Moreover, its validity needs to be tested in future large prospective series before potentially serving as a template for future database and research into patient outcomes. Finally, a potential limitation may be the high rate of patients with acute appendicitis enrolled in the study (42.1%). Some authors [18], after excluding patients with perforated appendicitis, found that the cure rate among patients who had peritonitis and were enrolled in clinical trials, was much higher than that of patients who were not enrolled and that the mortality rate was much lower. Although, delineating the source of infection as accurately as possible prior to surgery is described as the primary aim and the first step in managing acute peritonitis, in emergency departments of limited-resource hospitals, diagnosis of acute peritonitis is mainly clinical, and supported only by basic laboratory tests, and excluding acute appendicitis in the pre-operative phase would make the score impractical to a large part of the world’s population.

## Conclusions

This worldwide multicentre observational study was performed in 153 surgical departments from 56 countries over a 4-month study period (February 1, 2018–May 31, 2018). All consecutive patients admitted to surgical departments with clinical diagnosis of acute peritonitis were included in the study. The most significant independent variables associated with in-hospital mortality were adjusted to clinical criteria and were used to create a new bedside early warning score for patients with acute peritonitis. The simple PIPAS Severity Score for patients with acute peritonitis can be used on the global level and can help clinicians to assess patients with acute peritonitis at high risk for treatment failure and mortality. The authors created an acronym for the PIPAS Severity Score to help remember the variables “Scores Must Be Simple For Sepsis Risk Assessment” (severe cardiovascular disease, malignancy, blood oxygen saturation level, severe chronic kidney disease, fully alert, systolic blood pressure, respiratory rate, age).

## Data Availability

The authors are responsible for the data described in the manuscript and assure full availability of the study material upon request to the corresponding author.

## Apenndix

**Table 5:** PIPAS Severity Score for patients with acute peritonitis (range 0–8)

Variables	Score
Age (years)	
80 or more	1
Less than 80	0
Malignancy	
Yes	1
No	0
Severe cardiovascular disease	
Yes	1
No	0
Severe chronic kidney disease	
Yes	1
No	0
Respiratory rate ≥ 22 breaths/min	
Yes	1
No	0
Systolic blood pressure < 100 mmHg	
Yes	1
No	0
Blood oxygen saturation level (SpO_2_) < 90% in air	
Yes	1
No	0
AVPU responsiveness scale full alert	
No	1
Yes	0

## Abbreviations

AVPU: Alert/verbal/painful/unresponsive
COPD: Chronic obstructive pulmonary disease
CRP: C-reactive protein
CT: Computer tomography
INR: International normalised ratio
IQR: Interquartile range
LOS: Length of hospital stay
NRS: Numerical rating scale
PID: Pelvic inflammatory disease. IAIs: intra-abdominal infections
qSOFA: Quick Sequential Organ Failure Assessment
ROC: Receiver operating characteristic
US: Ultrasound
WBC: White blood count
WSES: World Society of Emergency Surgery
